# Long-term surveys of age structure in 13 ungulate and one ostrich species in the Serengeti, 1926–2018

**DOI:** 10.1038/s41597-020-00701-0

**Published:** 2020-10-21

**Authors:** Pierre Rogy, Anthony R. E. Sinclair

**Affiliations:** Department of Zoology and Biodiversity Research Centre, University of British Columbia, 6270 University Boulevard, Vancouver, British Columbia V6T 1Z4 Canada

**Keywords:** Population dynamics, Conservation biology

## Abstract

The Serengeti ecosystem spans an extensive network of protected areas in Tanzania, eastern Africa, and a UNESCO Wold Heritage Site. It is home to some of the largest animal migrations on the planet. Here, we describe a dataset consisting of the sample counts of three age classes (infant, juvenile and adult) of 13 ungulate and one ostrich species. Sample counts were tallied visually from the ground, or, in some instances, aerial photographs, during a period extending from 1926 to 2018. Observed animals were assigned to age classes based on specific criteria for each species. For nine of the 14 species of this dataset, the number of sampling years is over 30. This resulted in a total of 533 different records of count across age classes. By computing age-class ratios, these data can be used to measure long-term recruitment success at different ages of the tallied species. In particular, the temporal extent of these data allows comparison of patterns to other long-term processes, such as the El Niño-Southern Oscillation (ENSO).

## Abstract (summary)

**Table:** 

Measurement(s)	number of individuals in a given age class • Age Group • infant • juvenile • fully formed stage
Technology Type(s)	visual tallying • visual observation method • photography
Factor Type(s)	ungulate and ostrich species • year of data collection
Sample Characteristic - Organism	Syncerus caffer • Tragelaphus oryx • Loxodonta africana • Nanger granti • Eudorcas thomsonii • Giraffa camelopardalis • Aepyceros melampus • Alcelaphus buselaphus • Damaliscus lunatus • Phacochoerus africanus • Kobus defassa • Connochaetes taurinus • Equus burchellii • Struthio camelus
Sample Characteristic - Environment	terrestrial biome • savanna
Sample Characteristic - Location	Serengeti National Park

Machine-accessible metadata file describing the reported data: 10.6084/m9.figshare.12947528

## Background & Summary

Located in Tanzania, the Serengeti ecosystem (34–36 °E, 1–4 °S) has been a protected area since the 1920s, and is home to a great diversity of ungulate species. Over the past 150 years, this ecosystem has experienced several major perturbations that have affected the ecological dynamics of many these ungulate species. First, a panzootic due to the exotic rinderpest virus affected most ungulates, in particular wildebeest and buffalo, whose populations were decimated in the 1890s^1^. A very slow recovery followed, until the virus disappeared in 1963. After this local extirpation of the virus, these two species experienced exponential increase until numbers levelled out in 1977^2^. Second, intensive hunting has been an important threat to the ungulate populations. For example, in the mid-1800s, elephants were abundant in the ecosystem, but, by 1890, all had been killed for the ivory trade^3^. In 1951, when the Serengeti National Park was gazetted, greater conservation protection from poaching allowed recovery in all protected species^4^. In fact, it is only after 1950 that, through immigration of other populations from outside the core area, the elephant population of the Serengeti started a local resurgence, reaching a high point in 1975^3,5^. Following this, a second wave of ivory poaching reduced numbers to 10% of their previous high. In 1988, after the ban on ivory trading, poaching ceased and the elephant population rebounded to its highest density in 2014^6^. Ungulate populations of the ecosystem now face new threats^7^. Anthropogenic climate change induces acute modifications in precipitation patterns, particularly through variations in the El Niño-Southern Oscillations (ENSO)^8^. These changes are reflected in increasing fluctuations in vegetation patterns, which will ultimately influence the survival of ungulates^9,10^.

These examples illustrate that, in order to assess responses to human and environmental pressures, populations need to be monitored over the long run^11^. In ungulates, such responses have traditionally been measured through changes in reproductive success. This is usually accomplished by recording samples of two age groups of young animals relative to the number of females with which they are associated. In the case of the dataset presented here, the first age group, “infants”, indicates the early survival of young animals (less than six months of age, an unusual term used to make it applicable to both ungulates and ostrich) at the end of the rainy season (March–June). The second age group, “juveniles”, (between nine and 18 months of age, depending on the species) records the survival of young animals through the dry season, the period of food shortage.

This dataset consists of 13 species of ungulates: the African buffalo (*Syncerus caffer* Sparrman 1779), eland (*Taurotragus oryx* Pallas 1766), elephant (*Loxodonta africana* Blumenbach 1797), Grant’s gazelle (*Nanger granti* Brooke 1872), Thomson’s gazelle (*Eudorcas thomsonii* Günther 1884), giraffe (*Giraffa camelopardalus tippelskirchi* Brisson 1772), impala (*Aepyceros melampus* Lichtenstein 1812), Coke’s hartebeest or kongoni (*Alcelaphus buselaphus* Pallas 1766), topi (*Damaliscus lunatus* Burchell 1824), warthog (*Phacochoerus africanus* Gmelin 1788), Defassa waterbuck (*Kobus defassa* Rüppell 1835), wildebeest (*Connochaetes taurinus* Burchell 1823), and zebra (*Equus quagga* Boddaert 1785). In addition, this dataset includes similar measurements for the ostrich (*Struthio camelus* Linnaeus 1758). Samples were obtained by driving along roads and recording the age and sex of animals out to approximately 100 m, a distance where age classes can still be readily identified. For common species, listed in Method 1 below, this sampling was conducted once or twice a year at specific times, while, for rarer species, listed in Method 2 below, observations were *ad hoc* and all records for the year were summed. A special case of Method 1 were the very large herds of wildebeest (*C. taurinus*) and zebra (*Eq. quagga*), for which subsampling along transects were needed. Very early samples of African buffalo (*Sy. caffer*), giraffe (*G. camelopardalus*) and wildebeest (*C. taurinus*) were obtained from aerial photographs (Method 3, explained below). Although they do not provide a complete census of the populations, these data can be used to estimate rates of reproduction (# infants/ # adult females) or effective recruitment (# juveniles / # adult females) across the years of sampling.

## Methods

**Fig. 1:**
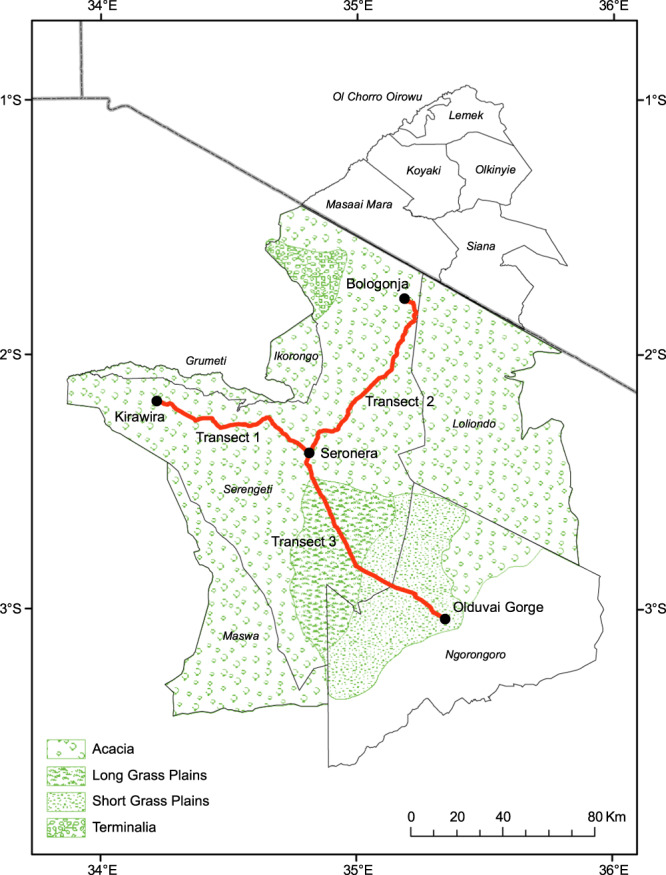
Ungulate and ostrich sampling transects in the Serengeti ecosystem.

There were three methods of sampling the populations. For Methods 1 and 2, records were obtained by driving along the road transects, and stopping to score the age groups in herds within some 100 m of the road. There were three road transects, entirely in the administrative boundaries of Serengeti National Park and consistent every year (1962–2018), with records summed over the three for each data entry. Transect 1 was from Seronera (34.823°E, 2.428°S) west to Kirawira (34.208°E, 2.151°S; 120 km), Transect 2 from Seronera to Bologonja (35.173°E, 1.757°S; 115 km), and Transect 3 from Seronera to Olduvai Gorge (35.35°E, 2.993°S; 75 km) (Fig. 1). The first two transects were in similar savanna ecosystems, and comparison of samples from these two showed close similarity.

The criteria for age classes in each species are given in Online-only Table 1. The sample was the herd within view (such as a group of impalas (*Ae. melampus*) or hartebeests (*Al. buselaphus*), which occur in discrete groups), or a subset of it if the herd was very large. One observer, using 8–10 x magnification binoculars, called out the age category while a recorder entered the records on data sheets. These were later entered digitally.

Two exceptions to this were the immense herds of migrant wildebeest (*C. taurinus*) and zebra (*Eq. quagga*). Because they were numerous and extensive, herds had to be sampled in a systematic way. A vehicle drove through the herds, stopping every half kilometer, where a 180 degree scan out to 100 m was conducted to count the sample within view. The transects were from the start to the end of the herd, with some being 30 km long through a single, continuous herd. Method 3 used aerial pictures of the herds to score age groups. Although the sampling protocol was different in the three methods (due to different distributions of each species) the same criteria for identifying age classes was used in all methods. All methods used either systematic or random sampling of the populations.

All species were either migrants, if the species shows seasonal variation in habitat, or residents, if the species remains in the same area of the park year-round. A notable exception to this is the wildebeest (*C. taurinus*). In fact, there were two populations of wildebeest, a large migrant herd and a small resident herd at the far western end of the ecosystem. These two were sampled separately and scored as either migrant or resident.

### Method 1

This method was used in all sampling years for impala (*Ae. melampus*), Coke’s kongoni (*Al. buselaphus*), topi (*D. lunatus*), warthog (*P. africanus*), Defassa waterbuck (*K. defassa*), and zebra (*Eq. quagga*). Sampling years 1984–1994 for African buffalo (*Sy. caffer*), 1965–2012 for giraffe (*G. camelopardalus*), and 1964–2016 for wildebeest (*C. taurinus*).

Populations were sampled once or twice a year at specific times, depending on the availability of different age classes in the areas near transects. Because ungulates had different birth seasons samples were collected at two time periods, once in mid-year and once at year-end. Only one time period per year was used for each species. The early age group, “infants”, was sampled usually near the end of the rainy season (March–June) since many species give birth during the rainy season. For some species, there was a second sampling period (August-December) at the end of the dry season, to measure the survival of juveniles during this period of ecological stress. There are a few cases where more than two samples were obtained in a single year, so as to track the survival of the whole cohort throughout a year.

### Method 2

This method was used in all sampling years for eland (*T. oryx*), elephant (*L. africana*), Grant’s gazelle (*N. granti*), ostrich (*S. camelus*), and waterbuck (*K. defassa*).

These species were sufficiently scarce that an adequate sample could not be obtained at specific times. For these, records were scored whenever the species was seen in a sampling period, and then records for all sampling periods of a single given year were summed. A special case was Thomson’s gazelle (*Eu. thomsonii*), which, although numerous, was scored only during one short time period (1992–1994) for the months of August and September.

### Method 3

This method was used in sample years 1965–1973 for African buffalo (*Sy*. caffer), and 1926–1933 for giraffe (*G. camelopardalus tippelskirchi*), wildebeest (*C. taurinus*), and zebra (*Eq. quagga)*. The area covered was in all cases within the Serengeti ecosystem. Buffalo and giraffe were only found in the savanna, while wildebeest were sampled when they were on the plains. Flights were made systematically over the area, wildebeest was sampled using photographs at regular intervals, buffalo and giraffe were sampled when they were encountered.

The third method, applied only in the very early years, used aerial photographs to identify age classes and females. The same criteria for identifying age classes was used as those for Methods 1 and 2 (Online-only Table 1), with an emphasis on the shape and size of horns for the wildebeest and African buffalo^2^, and of the relative sizes of young giraffe. The early samples in 1926–1933, were obtained from photographs taken by Martin Johnson. These photos reside in the Martin and Osa Johnson Safari Museum, Chanute, Kansas. Unfortunately, the 1965–1973 photographs of buffalo herds have now all been lost or destroyed.

## Data Records

The dataset includes 533 different year-month-species measurement, represented as a list of species names and their count in each age class. As shown in Table 1, the data consist of 15 columns comprising taxonomic information, count in each age class, as well as the information on sampling. The data are provided in a .txt file^12^.

**Table 1:** Description of dataset variables.

Variable	Description	Unit
order	Linnaean order of species	
family	Linnaean family of species	
genus	Linnaean genus of species	
specific_epithet	Linnaean specific epithet of species	
naming_authority	Scientific name authorship	
common_name	Common name of species	
year	Year of sampling	
month	Month of sampling	
infant	Count of infants	Individuals
juvenile	Count of juveniles	Individuals
female	Count of adult females	Individuals
unid_adult	Count of adult males and females (i.e. when adult males and females could not readily be differentiated)	Individuals
migrant_resident	Whether the population is migrant (“M”) or resident (“R”) in the ecosystem	
sampling_type	Which age classes were recorded for that specific sample: infants + juveniles + females (“ijf”), infants + juveniles + all adults (“ija”), infants + females (“if”), juveniles + females (“jf”), infants + all adults (“ia”), or juveniles + all adults (“ja”)	
sampling_method	Whether sampling Method 1 (sampling once or twice a year at specific times), 2 (yearly counts for rare species) or 3 (tallying using aerial pictures) was used for that specific sample	

## Technical Validation

Sampling of all herds seen along transects was designed to provide an unbiased measurement of recruitment success in the populations relative to the number of females. Therefore, males were not part of this sampling program for most species. This focus on females was important because males of many species separate from the female herds and become solitary or form bachelor herds. An unbiased sampling of males would therefore require an unmanageably large sample over the whole ecosystem. However, the sexes of two species, zebra (*Eq*. quagga) and warthog (*P. africanus*), could not be identified with certainty so males and females were recorded together as adults. In both of these species, males are evenly distributed with the females, so sampling remained relatively unbiased. This observation is based on a subset of data where the sexes could be distinguished and on published research^13^. In addition, although age is continuous, our age categories were discrete. This could induce bias when observed young are at the border of two age categories. However, the observers used consistent criteria to identify age classes (Online-only Table 1). In order to reduce the different individual biases that could arise from different observers, only one (A.R.E. Sinclair) scored the observations before 1997, including the photographs, and four observers conducted the survey between 1997 and 2018. All other observers were thoroughly trained by A.R.E. Sinclair.

## Data Availability

The code for compiling and generating the dataset is available in the Github repository for the project^14^.

## Online-only Table

**Online-only Table 1:** Criteria for identifying the different age and sex classes and for each species.

Species	Infant criteria	Infant age	Juvenile criteria	Juvenile age	Adult criteria
African buffalo (*Sy. caffer)*	Straight horns less than 15 cm (horns grow about 2 cm per month)	<6 months	Horns with tips turned inwards (in older animals the tips turn out again)	12–18 months	Females with thin horns, relative to males
Eland (*T. oryx*)	Straight horns less than 15 cm	<6 months	Straight horns less than 30 cm	6–12 months	Females not reliably separated from immature males, so both scored as adults
Elephant (*L. africana*)	Could walk underneath the mother	<3 months	Between infants but smaller than height of the mother’s elbow	12–18 months	Females identified by mammary glands and genitalia
Giraffe (*G. camelopardalus tippelskirchi*)	Head did not reach the hump at base the mother’s neck	<6 months	Head above hump on the mother’s neck but not higher than halfway up neck	12–18 months	Females identified by lack of a forehead lump, and by genitalia
Grant’s gazelle (*N. granti*)	Head not as high as the mother’s back	<3 months	Head just above the mother’s back	<6 months	Females with thin horns and short, relative to males
Thomson’s gazelle (*Eu. thomsonii*)	Head not as high as the mother’s back	<3 months	Head just above the mother’s back	<6 months	Females with thin horns and short, relative to males
Impala (*Ae. melampus*)	Head not as high as the mother’s back	<4 months	Head just above the mother’s back	5–12 months	Females identified by lack of horns
Coke’s kongoni (*Al. buselaphus*)	Straight horns less than 10 cm (horns grow about 2 cm per month)	<6 months	Horns between 10 cm and tips turned inwards (in older animals the tips turn out again)	12–18 months	Females with thin horns, relative to males
Ostrich (*St. camelus*)	Less than 50 cm in height	<3 months	Between 50 cm and halfway up neck of females	1–2 years	Females of different size and color than males
Topi (*D. lunatus*)	Straight horns less than 10 cm (horns grow about 2 cm per month)	<6 months	Horns between 10 cm and tips turned inwards (in older animals the tips turn out again)	12–18 months	Females with thin horns, relative to males
Warthog (*P. africanus*)	Head not as high as the mother’s back	<4 months	Head just above the mother’s back	<9 months	Sexes only identifiable by genitalia, often not visible in long grass, hence both scored together as adults
Defassa waterbuck (*K. defassa*)	Head not as high as the mother’s back	<4 months	Head just above the mother’s back	5–12 months	Females identified by lack of horns
Wildebeest (*C. taurinus*)	Straight horns less than 10 cm (horns grow about 2 cm per month)	<6 months	Horns between 10 cm and tips turned inwards (in older animals the tips turn out again)	12–18 months	Females with thin horns, relative to males
Zebra (*Eq. quagga*)	Head not as high as the mother’s back	<6 months	Head just above the mother’s back	12–18 months	Sexes only identifiable by genitalia, often not visible in long grass, hence both scored together as adults
